# Diagnosis and Treatment of Peritalar Injuries in the Acute Trauma Setting: A Review of the Literature

**DOI:** 10.1155/2020/1852025

**Published:** 2020-01-03

**Authors:** Abdul R. Arain, Curtis T. Adams, Stefanos F. Haddad, Muhammad Moral, Joseph Young, Khusboo Desai, Andrew J. Rosenbaum

**Affiliations:** Albany Medical Center, Albany, NY, USA

## Abstract

The bony and ligamentous structure of the foot is a complex kinematic interaction, designed to transmit force and motion in an energy-efficient and stable manner. Visible deformity of the foot or atypical patterns of swelling should raise significant concern for foot trauma. In some instances, disruption of either bony structure or supporting ligaments is identified years after injury due to chronic pain in the hindfoot or midfoot. This article will focus on injuries relating to the peritalar complex, the bony articulation between the tibia, talus, calcaneus, and navicular bones, supplemented with multiple ligamentous structures. Attention will be given to the five most common peritalar injuries to illustrate the nature of each and briefly describe methods for achieving the correct diagnosis in the context of acute trauma. This includes subtalar dislocations, chopart joint injuries, talar fractures, navicular fractures, and occult calcaneal fractures.

## 1. Introduction

Fractures and ligamentous injuries of the foot are significant and challenging entities in the context of orthopaedic trauma [1]. Foot fractures are regarded as the most frequently missed extremity fracture [2]. Similarly, ligamentous injuries and related dislocations present many diagnostic challenges. There are multiple potential causalities underlying these missed injuries, which will be discussed in detail.

In the acute trauma patient, priority is given to executing ATLS protocol and emergent resuscitation. Hemodynamically unstable patients necessitate immediate life-saving measures while stable patients can undergo full clinical and radiologic workup to identify less obvious injuries.

In the resuscitated patient, priority is initially directed toward open injuries, fractures causing neurologic impairment, or injuries at high risk for compartment syndrome. Beyond this, a thorough primary survey with direct examination and palpation of every joint and extremity is critically important, and its importance cannot be overstated. The physical exam should further be correlated with radiographic evaluation of any site of suspected fracture, utilizing stress tests where appropriate and maintaining high clinical suspicion for particular fracture patterns based on the mechanism of injury.

In recent years, lisfranc injuries have been discussed extensively due to the frequency with which they are missed and the high probability for requiring operative treatment [3, 4]. In contrast, hindfoot injuries, particularly peritalar fractures and dislocations, have received significantly less attention. Both are relatively rare in the context of orthopaedic trauma; however, the previous literature has demonstrated a significant increase in morbidity of polytraumatized patients with an untreated foot injury [5]. Within the category of peritalar injuries, the most common missed injury is a fracture of the talus. Together with occult fractures of the calcaneus and navicular, these form roughly 70% of all missed foot injuries in high and low energy trauma [3].

Previous research has identified several signs useful in identifying occult foot fractures [4]. These include pain out of proportion to provisional diagnosis, which suggests a radiographically invisible fracture or ligamentous injury. Visible deformity of the foot or atypical patterns of swelling also raise significant concern. In the long-term, one should suspect missed foot injury when there is failure of symptoms to improve in the days and weeks following the initial trauma. Finally, a high-energy or classically described mechanism, such as an axial load on a plantar-flexed foot, should raise serious concern for an occult injury.

## 2. Peritalar Complex

The peritalar complex is a bony articulation between the tibia, talus, calcaneus, and navicular bones, supplemented with multiple ligamentous structures. Within this complex, the talar-navicular interaction, coupled with the spring ligament, is most important for maintaining integrity of the medial column [6]. The peritalar joints have been described to consist of two separate, independently functioning capsules involving the subtalar and talocalcaneonavicular joints, respectively [7]. Recent studies have emphasized the complex anatomic and kinematic relationship between the talocalcaneal and talonavicular joints and their contributions to hindfoot function [8].

In its uninjured state, the peritalar complex carries out several critical functions. The talus, in its relation between the calcaneus and tibia, acts to transmit a valgus force upon heel strike from the laterally-oriented calcaneus through the talus to the mid- and forefoot. This thrust unlocks the transverse tarsal joints and enables the talus to transmit directional torque away from the tibiotalar joint, thereby decreasing tibiotalar tilt stress. Hence, the subtalar joint functions to convert the foot from a mobile structure at heel strike to a rigid structure at toe off [7]. The talonavicular joint, supported by the plantar spring ligament complex of the calcaneonavicular ligaments, is the most important contributor of medial column stability. Additionally, the spring ligament supports the head of the talus and acts as a primary static restraint at the talonavicular joint to prevent excursion [9].

Peritalar injuries have been defined as fractures or ligamentous disruption resulting in instability of one or more peritalar joints: tibiotalar, subtalar, calcaneocuboid, and talonavicular [3]. As discussed, the bony and ligamentous structure of the foot is a complex kinematic interaction, designed to transmit force and motion in an energy-efficient and stable manner. Disruption of either bony structure or supporting ligaments results in long-term functional deficits, sometimes only identified years after injury by chronic pain in the hindfoot or midfoot due to progressive wear and strain.

The following sections will review the five most common peritalar injuries in order to illustrate the nature each and briefly describe methods for achieving the correct diagnosis in the context of acute trauma.

## 3. Subtalar Dislocation

**Figure 1:**
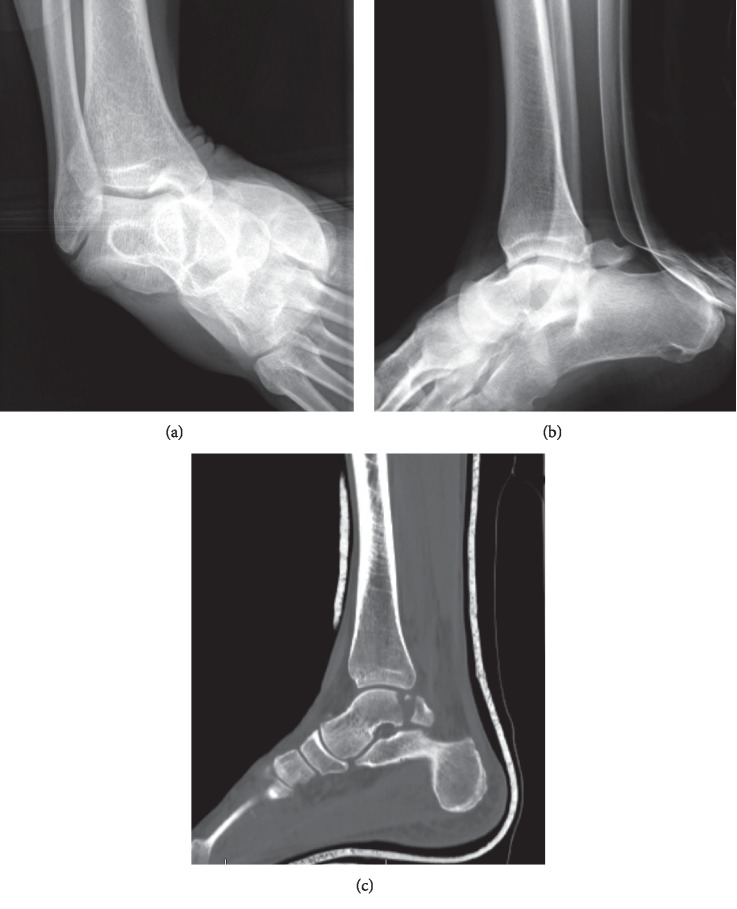
AP (a) and lateral (b) ankle X-rays demonstrating a medial subtalar fracture dislocation. The talar dome remains in normal articulation with the ankle joint, with the ankle mortise intact. Postreduction CT (c) reveals a coronally oriented, comminuted, and displaced fracture of the posterior talar dome and posterior talar process, with intraarticular extension into the subtalar joint.

Subtalar joint stability is primarily dependent on ligamentous structures, as described above. Forceful inversion, eversion, or extreme plantar flexion may cause ligamentous failure resulting in traumatic displacement of the calcaneus and navicular. Isolated subtalar dislocations are rare and more often present with associated fractures of the malleoli, fifth metatarsal, or talus (Figure 1). Patients with ligamentous insufficiency, malleolar hypoplasia, or other peritalar deformity are at increased risk of sustaining subtalar dislocation [4].

Subtalar dislocations almost invariably present as either grossly dislocated or spontaneously reduced. In the case of gross dislocation, there is visible deformity with skin tension on the opposing side. The amount of force required frequently results in a substantial skin defect on the tension side of the wound, creating an open subtalar dislocation. Conversely, many subtalar dislocations spontaneously reduce, leaving no radiographic sign of associated fractures. These patients present with significant soft tissue swelling and ecchymosis of the mid- and hindfoot, often similar to an ankle sprain. MRI studies may be helpful in cases of high clinical suspicion for a spontaneously reduced subtalar dislocation.

Broca was among the first to suggest a classification scheme for subtalar dislocations, describing medial, lateral, and posterior dislocations of the calcaneus and foot from beneath the talus [10]. Later, it was noted that anterior dislocations can occur; however, these are exceedingly rare. Medial dislocations are most common in the context of lower extremity trauma. Forceful inversion of the forefoot in a plantarflexed position loads the lateral collateral ligaments, resulting in rupture of the talocalcaneal and talonavicular ligaments and pivot of the talus on the sustentaculum tali. Conversely, lateral dislocations are caused by traumatic eversion of the foot and posterior dislocation by forceful plantar flexion alone [11].

Treatment of subtalar dislocations is highly dependent on skin integrity and associated fracture patterns. In cases of isolated ligamentous injury with intact skin, closed reduction and immobilization is almost universally recommended. Previously literature supports both immobilization for greater than 4 weeks, and early protected range of motion exercises with the goal of avoiding subtalar stiffness [11]. Lateral dislocations carry a poorer prognosis overall, in part due to the higher energy force necessary to cause sufficient eversion of the foot and dislocation of the calcaneus and navicular [12]. Significant soft tissue injury and skin defects overlying the site of dislocation should be treated as an open injury, requiring formal debridement, irrigation, reduction, closure, and course of parenteral antibiotics.

Complications following subtalar dislocation are well described in the literature. Posttraumatic arthritis is noted in up to 80% of patients and may present in the talonavicular, tibiotalar, or talocalcaneal joints. Osteonecrosis of the talus has also been described as a late complication of subtalar dislocation. Finally, subtalar joint stiffness is commonly observed as a result of fibrosis of the joint capsule following injury [3]. Newer evidence supports shorter-term immobilization followed by early range of motion after the initial injury in order to prevent stiffness [11].

## 4. Chopart Joint Injury

The chopart joint consists of the combined talonavicular and calcaneocuboid joints. The talonavicular joint allows for pronation and supination of the foot as part of the talocalcaneonavicular joint. The calcaneocuboid joint lies in the lateral column and provides both flexibility and suspension to the foot.

Chopart joint injuries may be purely ligamentous or combined fracture and ligamentous injury. In both cases, there is loss of stability across the transverse tarsal joints. These injuries may occur in conjunction with a talar head, navicular, or cuboid fracture, which should raise suspicion for an associated ligamentous injury [8].

Chopart joint injuries are uncommon and may be difficult to detect clinically. Kou and Fortin recommend evaluating joint space asymmetry across the transverse tarsal joints on foot radiographs [3]. This may reveal a ligamentous disruption, especially in the setting of soft tissue swelling and midfoot pain. We recommend manual stress or weightbearing films with contralateral comparison in order to highlight ligamentous insufficiency of the talonavicular or calcaneocuboid joints.

The treatment of Chopart joint injuries is largely based on case reports and individual surgeon preference, given the relative paucity of the literature. Initial management begins with immobilization of the joint. In cases of refractory pain or continued tarsal instability, transverse tarsal fusion may be necessary [13]. The use of bilateral stress films is helpful in identifying instability and guiding surgical management. In cases of minimal instability identified on stress views, temporary percutaneous fixation is often sufficient to stabilize the anatomic joint. Malreduction or gross deformity may require open reduction and internal fixation. As a last resort, salvage arthrodesis may prevent midfoot collapse and resultant loss of function. A previous literature has demonstrated that fusion of the calcaneocuboid joint may provide indirect stability of the talonavicular joint, and results in less functional impairment given the inherent flexibility of the lateral column [14]. Following reduction and/or fixation of Chopart joint injuries, weight bearing is generally restricted for 8–10 weeks following injury, with graduated range of motion exercises and slow return to activity thereafter.

## 5. Talar Fracture

**Figure 2:**
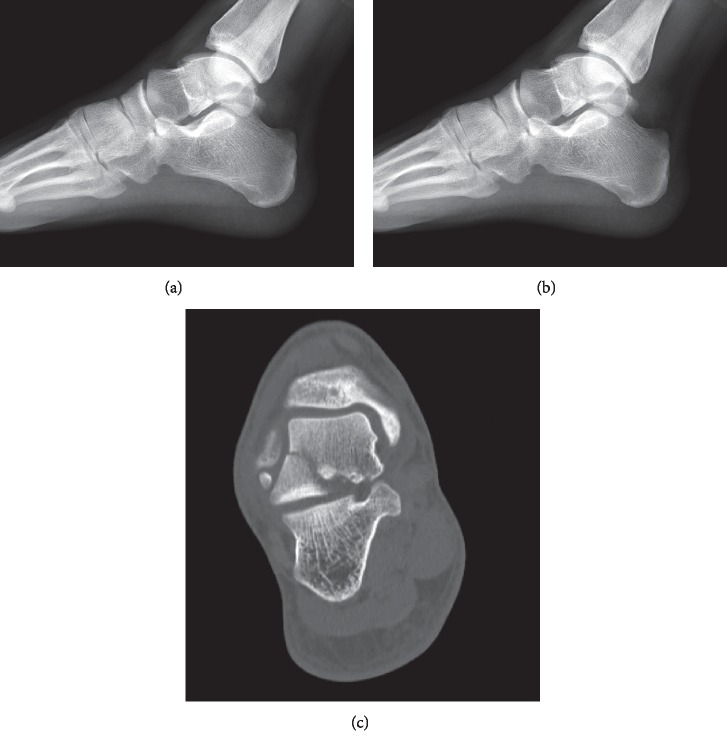
Lateral foot X-ray (a) demonstrating subtle fracture, vertically oriented across the talar body. Sagittal (b) and coronal (CT) cuts better elucidating fracture pattern. Primary fracture line extends posterior to the lateral process of the talus. There is intraarticular extension into the middle and posterior subtalar joints. Fracture extends into the anterior talar dome with articular step-off.

**Figure 3:**
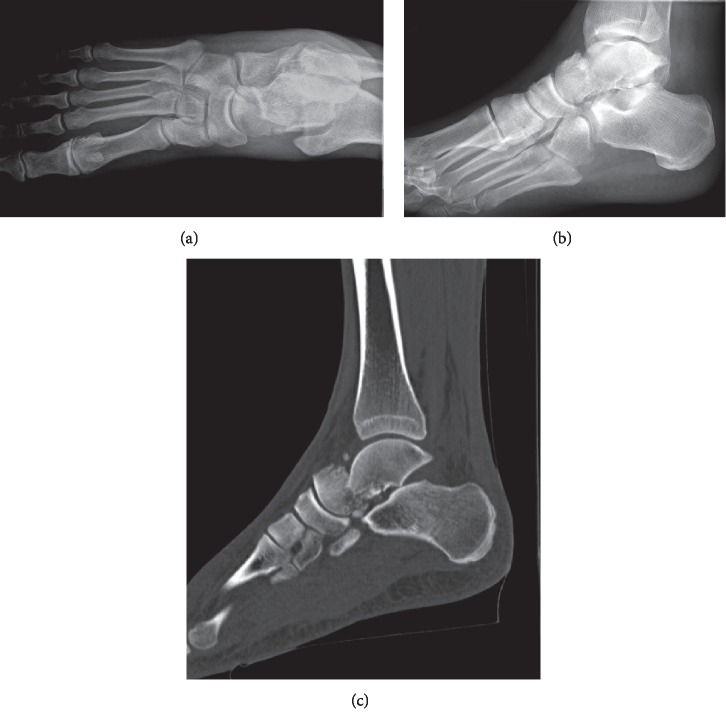
AP (a) and lateral (b) foot X-rays demonstrating a displaced talar neck fracture. CT scan (c) better elucidates the comminution and fracture pattern.

Multiple fracture patterns of the talus have been described in their relation to peritalar injuries (Figures 2 and 3). These include fractures of the talar head and neck as well as of the lateral and posterior processes [15]. In addition, occult talar dome injury may be present. In each case, as with all peritalar injuries, findings range from radiologically benign to grossly displaced fragments. Pain out of proportion to provisional diagnosis can be key to further investigating a possible talar fracture.

Fracture of the talar head is relatively rare, with a described incidence of <10% of talar fractures. This pattern is caused by a combined dorsiflexion and inversion force that longitudinally loads the talus, resulting in a shear fracture across the talar head [16]. As a result, the medial column is functionally shortened, often resulting in a cavovarus foot with abnormal loading of the lateral column. Patients often present after a high-energy motor vehicle accident or fall. Clinically they demonstrate soft tissue swelling at the midfoot, variable gross deformity, ecchymosis, and significant pain on palpation or range of motion. Gross ecchymosis often signifies injury beyond a simple sprain. Radiographic findings are variable; therefore, CT scans of the foot are most helpful in diagnosis and treatment planning.

Treatment of talar head fractures varies from conservative immobilization to percutaneous pinning and to open reduction and internal fixation of displaced fragments [16]. In cases of late or missed diagnosis, salvage arthrodesis may be an additional option. Medial column shortening may also require distraction and bone block fusion. Avascular necrosis, although commonly described in talar neck fractures, is rare in the setting of talar head fracture due to adequate blood supply and multiple ligamentous attachments.

Similar to the talar head, lateral process fractures are often missed due to subtle or absent radiographic findings. Commonly referred to as “snowboarder's ankle,” the lateral process comprises up to 24% of talar body fractures [17]. The mechanism of injury involves a dorsiflexed, inverted foot subjected to an external rotatory force, commonly due to high energy forces. Clinically, lateral process fractures can be misdiagnosed as ankle sprains of the anterior tibiofibular ligament, given the similarity of presentation and site of localized pain [17]. Radiographically, ankle mortise views provide a profile of the lateral process. CT scan remains the imaging modality of choice and frequently reveals more extensive comminution and displacement than originally evident on radiographs.

Unlike the talar head, lateral process fractures are typically classified as intraarticular injuries. These fractures extend to the facets that articulate with the distal fibula and the anterolateral subtalar joint. As a result, missed diagnoses pose increased risk of significant subtalar arthritis [5]. Similar to talar head fractures, nondisplaced lateral process fractures may be treated with immobilization and graduated weight bearing, while open reduction and internal fixation is appropriate where there is articular comminution or displacement. Chronic pain after a missed, remote injury may necessitate subtalar fusion if significant joint arthrosis is present.

Fractures of the posterior process of the talus involve a larger portion of the talar body, roughly 25% of the articulating subtalar surface [3]. Like other talar fractures, these typically involve high energy mechanisms. Avulsion of the lateral tubercle can occur through forceful inversion, whereas extreme plantar flexion crushes the posterior malleolus against the posterior process of the talus. Similarly, the medial tubercle of the posterior process may avulse through tension of the deltoid ligament in forceful dorsiflexion with pronation. Clinically, patients experience pain with forced plantar flexion as the posterior process of the talus is compressed. Location of pain is dependent on the location of the fracture within the posterior process; fragments that disrupt the groove for the flexor hallicus longus often elicit pain with great toe flexion and extension. Fractures that involve the posteromedial tubercle are universally accompanied by medial subtalar joint dislocation and frequent tenderness to palpation over the medial posterior ankle [11].

On radiographs, posterior process fractures must be distinguished from an intact os trigonum, a secondary talar ossification center. If present, the os trigonum is located posterior to the lateral tubercle and may resemble a fracture fragment. As with other talar fractures, a CT scan remains the most reliable method of identifying subtle fractures, especially in the presence of suggestive clinical symptoms.

Displaced posterior process fractures are treated via open reduction and internal fixation, while nonsurgical treatment may be judiciously employed for nondisplaced, minimally comminuted fractures. In the presence of significant displaced comminution, surgical removal of fragments may improve long-term outcomes. As with other talar fractures, subtalar arthrodesis may be required for post-traumatic subtalar arthrosis.

## 6. Navicular Fracture

**Figure 4:**
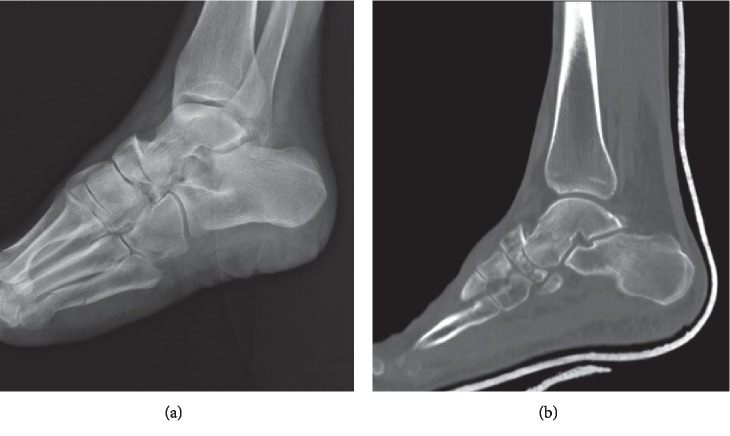
Lateral foot X-ray (a) demonstrating talonavicular fracture dislocation. In addition, there are fractures of the 4th and 5th metatarsal shafts. CT scan (b) better elucidates the fracture pattern. The talus was driven into the lateral aspect of the navicular producing a comminuted fracture.

Fractures of the tarsal navicular occur when the talus and the cuneiforms of the midfoot compress the navicular (Figure 4). This combination of forces most often results in transverse fractures towards the plantar aspect of the navicular, resulting in superior displacement of the dorsal navicular fragment [9]. As a result, the medial column is fundamentally damaged and frequently demonstrates gross instability. Clinically, navicular fractures may present with gross deformity, such as tenting of the skin overlying a displaced navicular fragment, or patients may only endorse midfoot pain and swelling. Plain films should be taken to evaluate the continuity between the dorsal and plantar aspects of the navicular and associated cuneiforms; displacement is indicative of fracture. CT scans are most useful in determining whether there is plantar comminution. Treatment in the acute setting consists of immobilization and protected weight bearing for nondisplaced fractures, with open reduction and internal fixation if displacement is present. Consideration in either case must be given to the propensity of the navicular for nonunion or avascular necrosis, given its limited blood supply. As with talar fractures, chronic pain after remote navicular fracture can be treated with talonavicular arthrodesis.

## 7. Occult Calcaneal Fracture

The calcaneus is the largest tarsal bone in the foot and the most commonly fractured. There are several major fracture variants which present with obvious clinical or radiographic findings; however, in the context of traumatic peritalar injuries, two important subtler fracture patterns can be difficult to identify [3]. A Sanders Type II fracture with a lateral calcaneal fragment involving the calcaneal tuberosity displaced laterally and proximally may result in lateral dislocation of the subtalar joint. Unlike the majority of high energy calcaneal fractures, this variant has been shown to result from lower energy axial, twisting forces. This lateral calcaneal displacement is often difficult to distinguish on AP and lateral radiographs but may be visible on a mortise view of the ankle, where the displaced lateral calcaneal facet appears beneath the distal fibula. Open reduction and internal fixation provides definitive treatment, with subtalar fusion for posttraumatic subtalar arthrosis [18].

A rare calcaneal variant is an isolated sustentaculum tali fracture, where the Bohler angle is again preserved [3]. In contrast to a Sanders Type II fracture, this variant involves high energy axial forces with varus loading and rotation, a mechanism which can produce concomitant talus fractures. Clinical suspicion in the presence of hind or midfoot swelling, ecchymosis, or unexplained pain should prompt thorough evaluation, with CT imaging if there is concern for fracture not apparent on plain films. As with the majority of calcaneal fractures, open reduction and internal fixation is the treatment of choice for sustentacular fractures.

## 8. Summary

In the context of trauma, with frequent concomitant large bone injury, neurologic deficit, or hemodynamic instability, injuries of the foot can prove both less urgent and more difficult to treat. Once a patient is stabilized and, however, other injuries have been addressed, appropriate orthopaedic care should be undertaken of all extremities. Given the frequency of tarsal bone injury in the context of high energy trauma, attention must be given to identifying possible fractures, ligamentous injuries, and dislocations of the hind and midfoot.

A high degree of clinical acumen and suspicion for injury must be employed in the presence of significant soft tissue swelling, hind or midfoot ecchymosis, gross deformity, crepitus, instability, or pain out of proportion to the provisional diagnosis. Standard foot X-rays along with oblique views should be obtained to evaluate for fractures. We also recommend obtaining contralateral foot films to thoroughly assess for occult injuries and posttraumatic changes. When initial films are unrevealing, CT scanning is invaluable in evaluating the degree of comminution and displacement of known fractures. It can also help with operative planning and assessment of union following nonoperative management of known injuries.

A thorough understanding of peritalar injuries is important as a high degree of morbidity results if the injury is missed. The most common consequence is chronic pain and progressive arthrosis, often necessitating salvage arthrodesis. As we have noted above, several fracture variants, including chopart joint injury and occult calcaneal fractures, are mentioned primarily in case reports. Further research of peritalar injuries should include more thorough retrospective reviews of these fractures.
